# Complete genome sequence of a *Streptococcus equi* subsp. *equi* bacteriophage isolated from raw sewage water in Tokyo

**DOI:** 10.1128/mra.01157-25

**Published:** 2026-03-05

**Authors:** Kikuno Shirakawa, Keiko Kawamoto, Kohei Kondo, Shinjiro Ojima, Yuta Kinoshita, Hidekazu Niwa, Michiyo Kataoka, Tadaki Suzuki, Takanori Ueno, Yo Sugawara, Koichi Watashi, Motoyuki Sugai, Kotaro Kiga, Yusuke Sato'o

**Affiliations:** 1 Laboratory of Infection Control and Immunology, Faculty of Veterinary Medicine, Azabu University 47710, Sagamihara, Kanagawa, Japan; 2 Antimicrobial Resistance Research Center, National Institute of Infectious Diseases, Japan Institute for Health Security, Higashimurayama, Tokyo, Japan; 3 Department of Drug Development, National Institute of Infectious Diseases, Japan Institute for Health Security 739298 , Shinjuku, Tokyo, Japan; 4 Microbiology Division, Equine Research Institute, Japan Racing Association 68373, Shimotsuke, Tochigi, Japan; 5 Department of Infectious Disease Pathology, National Institute of Infectious Diseases, Japan Institute for Health Security 739298 , Shinjuku, Tokyo, Japan; 6 Department of Infectious Disease Pathobiology, Graduate School of Medicine, Chiba University 12737, Chiba, Japan; Portland State University, Portland, Oregon, USA

**Keywords:** bacteriophage, *Streptococcus equi *subsp. *equi*, strangles

## Abstract

We have determined the complete genome sequence of a bacteriophage infecting *Streptococcus equi* subsp. *equi*, the causal agent of strangles in members of the family Equidae. This phage was isolated from wastewater and shows high similarity to C1 phage infecting other *Streptococcus* species.

## ANNOUNCEMENT

Drug-resistant bacteria pose a significant and rising threat (1, 2). Phage therapy is one potential strategy to address this issue (3), and its application in horses has been previously assessed (4–6). Strangles, an infectious upper respiratory condition, is caused by *Streptococcus equi* subsp. *equi* (7). An epidemiological survey recently reported that some strains have acquired resistance against key therapeutic agents (8). These circumstances highlight the exigency of isolating candidate bacteriophages for phage therapy.

**Fig 1:**
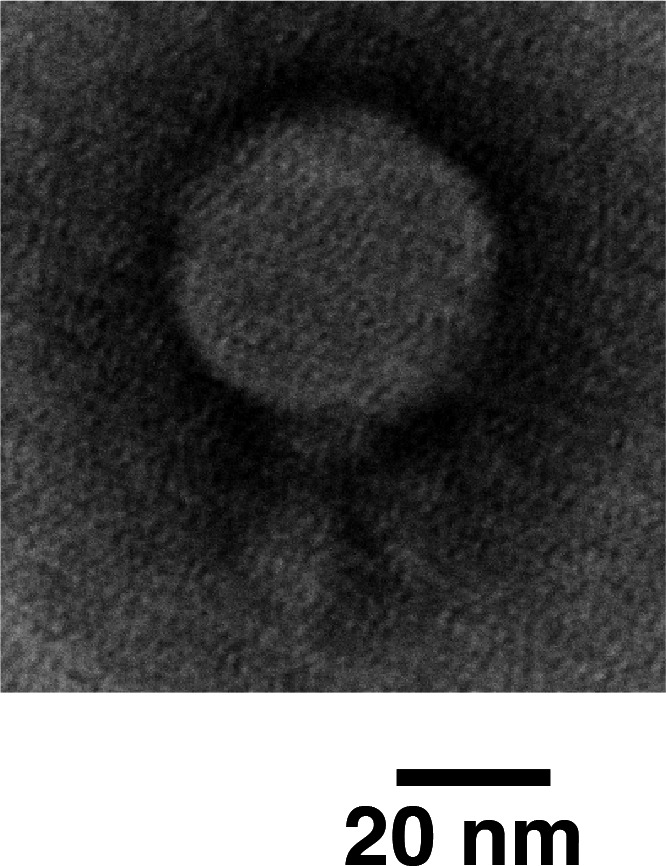
A transmission electron micrograph of purified virions, prepared using an Amicon Ultra-15 filter (100 K), showing an icosahedral head and short non-contractile tail characteristic of podophages.

We isolated phage from wastewater collected from a sewage plant in Tokyo in June 2023, then centrifuged and filtered through a 0.45-µm filter. Phage amplification was performed using brain heart infusion (BHI) broth (Difco, MI, USA) supplemented with 10 mM CaCl₂ and 10 mM MgSO₄, with *S. equi* ATCC 33398 used as host bacterium. Cultures were incubated at 22°C–24°C for 3 days. In this step, 62.5% of the final culture volume was the sewage sample, and 6.25% was the preculture inoculum. After amplification, single clear plaques were observed on ATCC 33398 lawns. A single phage was isolated by sub-culturing and designated vB_SEEA3_AUVM_23KS1. Transmission electron microscopy using an HT7700 (Hitachi Ltd., Tokyo, Japan), performed following a previously described method (9), revealed that the phage possesses Podoviridae-type morphology (Fig. 1).

Genomic DNA was extracted from virions purified with an Amicon Ultra-15 filter (100K; Millipore, Billerica, MA, USA), followed by purification using a QIAamp kit (Qiagen, Hilden, Germany). A library was prepared using Enzymatics 5× WGS Fragmentation Mix and WGS Ligase reagents (Qiagen) according to the manufacturer’s protocol and sequenced using an Illumina NovaSeq X Plus sequencer (Illumina, CA, USA). A total of 2,237,347 paired-end reads (2 × 151 bp) were assessed with FastQC (https://github.com/s-andrews/FastQC) and assembled using Shovill v1.1.0 (https://github.com/tseemann/shovill), yielding an average of 585.5-fold coverage. Sanger sequencing was performed to verify low-confidence regions at both ends of the sequence, using an Applied Biosystems 3730xl DNA Analyzer (Thermo Fisher Scientific, MA, USA). A final consensus sequence was obtained by integrating the NGS and Sanger data, resulting in a complete genome, which was annotated using Pharokka (10). Geneious Prime version 2025.2.2 (Biomatters Ltd., Auckland, New Zealand) was used for genomic comparisons. All tools were used with default parameters.

The genome of vB_SEEA3_AUVM_23KS1 is 16,051 bp in length, with 33.5% GC content and 23 open reading frames. Termini were determined by contig circularization. This phage shares 88.9% nucleotide identity with the *Fischettivirus* phage C1, which is propagated in group C *Streptococcus; S. equi* also belongs to this same group (11, 12). The gene corresponding to ORF20 in the C1 phage is missing, as are terminal repeats. This phage also shares 34.0% nucleotide identity with phiSG005 isolated in Japan from *Streptococcus gordonii* (13). These findings tend to indicate that the newly isolated phage diverged from a common ancestor shared with these phages, and the observed differences are assumed to represent long-term evolutionary changes associated with their respective host species (14). Collectively, these data suggest that vB_SEEA3_AUVM_23KS1 has evolved in parallel with the currently underway genetic contraction of the bacterium, which has reportedly undergone lineage-restricted evolutionary bottlenecks in recent history (14).

## Data Availability

The accession and BioSample numbers of the isolated phage are LC890459 and SAMD01681848, respectively. Raw data are deposited in DRR893225 (Sequence Read Archive) and LC909108, LC909109, LC909110, LC909111, and LC909112 (Sanger sequencing data).
